# Atrazine Toxicity: The Possible Role of Natural Products for Effective Treatment

**DOI:** 10.3390/plants12122278

**Published:** 2023-06-12

**Authors:** Srijit Das, Hussein Sakr, Isehaq Al-Huseini, Raghu Jetti, Sara Al-Qasmi, Raju Sugavasi, Srinivasa Rao Sirasanagandla

**Affiliations:** 1 Department of Human and Clinical Anatomy, College of Medicine and Health Sciences, Sultan Qaboos University, Muscat 123, Oman; s.das@squ.edu.om; 2 Department of Physiology, College of Medicine and Health Sciences, Sultan Qaboos University, Muscat 123, Oman; hsakr@squ.edu.om (H.S.); isehaq@squ.edu.om (I.A.-H.); 3 Department of Basic Medical Sciences, College of Applied Medical Sciences, King Khalid University, Abha 62521, Saudi Arabia; raghujetti82@gmail.com; 4 College of Medicine and Health Sciences, Sultan Qaboos University, Muscat 123, Oman; sara.alqasmi@hotmail.com; 5 Department of Anatomy, Fathima Institute of Medical Sciences, Kadapa 516003, India; anatraju81@gmail.com

**Keywords:** atrazine, toxicity, natural products, natural compounds, drug design, treatment

## Abstract

There are various herbicides which were used in the agriculture industry. Atrazine (ATZ) is a chlorinated triazine herbicide that consists of a ring structure, known as the triazine ring, along with a chlorine atom and five nitrogen atoms. ATZ is a water-soluble herbicide, which makes it capable of easily infiltrating into majority of the aquatic ecosystems. There are reports of toxic effects of ATZ on different systems of the body but, unfortunately, majority of these scientific reports were documented in animals. The herbicide was reported to enter the body through various routes. The toxicity of the herbicide can cause deleterious effects on the respiratory, reproductive, endocrine, central nervous system, gastrointestinal, and urinary systems of the human body. Alarmingly, few studies in industrial workers showed ATZ exposure leading to cancer. We embarked on the present review to discuss the mechanism of action of ATZ toxicity for which there is no specific antidote or drug. Evidence-based published literature on the effective use of natural products such as lycopene, curcumin, *Panax ginseng*, *Spirulina platensis*, Fucoidans, vitamin C, soyabeans, quercetin, L-carnitine, *Telfairia occidentalis*, vitamin E, *Garcinia kola*, melatonin, selenium, *Isatis indigotica*, polyphenols, *Acacia nilotica*, and *Zingiber officinale* were discussed in detail. In the absence of any particular allopathic drug, the present review may open the doors for future drug design involving the natural products and their active compounds.

## 1. Introduction

Herbicides, also defined as weed killers, are substances that are commonly used worldwide to control weeds and help increase agricultural yield [1]. However, the aimless use of these herbicides to improve agricultural production may have effects on the environment as well as on humans and animals. ATZ (2-chloro-4-ethylamino-6-isopropylamino-s-triazine) is the most frequently used herbicide found in agricultural environments [1]. This herbicide is widely applied on several agricultural crops, corn, sorghum, sugarcane, pineapples, and, to a lesser extent, on the landscape vegetation [1]. The herbicide may be commonly used in the form of sprays, liquids, concentrates, or in granular form. ATZ is commonly detected in many places which has an accumulation of water such as ponds, wells, ground surface, or even underground.

ATZ was first introduced in 1958 and is commonly used for the control of weeds in crops [2]. ATZ is a chlorinated triazine herbicide that consists of a ring structure, known as the triazine ring, along with a chlorine atom and five nitrogen atoms [3]. Modern herbicides are often synthetic and do not occur naturally. Pure ATZ is a white, odorless, and often colorless powder, rarely volatile, flammable, or reactive. The availability of ATZ ranges from granular and ready-to-use formulation, dissolved emulsifiable concentrates, and wettable powder [3]. It can be introduced into the air through vaporization, with a predicted half-life of 14 h [4]. Moreover, it is predominantly removed from the air by rainfall [4].

### 1.1. Atrazine Toxicity

ATZ is among the most commonly identified herbicides in the aquatic areas in United States [5]. ATZ is categorized as a restricted use pesticide by the United States Environmental Protection Agency (EPA) [5]. According to EPA norms, its maximum contamination limit (MCL) was set at a concentration of 3 ppb or 0.003 µg/L [5]. The 1991 European Union pesticide regulation adopted a stricter stance and restricted the use of substances considered harmful to the environment, groundwater, or human health [5].

ATZ is a water-soluble herbicide, which makes it capable of easily infiltrating into most aquatic ecosystems. Additionally, careless repeated applications of ATZ resulted in large quantities of the herbicide being precipitated and finding its way into water bodies [6]. Two studies from China reported concentrations of ATZ in the soil and groundwater of 97% and 89%, respectively [7]. A study conducted in the year 2020 to determine the ATZ levels in 1135 lakes and reservoirs in 48 states in the US revealed that ATZ was identified in about 32% of waterbodies, at a mean concentration of 0.17 μgL^−1^ [8]. Reports available from Mexico and Venezuela showed that the ATZ levels were found in the range of 5.77 to 402.00 ng L^−1^ and 1.00–1990 ngL^−1^, respectively, in the surface water [9]. It was initially thought that, since its mode of action is to prohibit photosynthesis in the desired plants, it would spare other species from its deleterious effects. Unfortunately, it was soon suspected that ATZ can indirectly and directly affect aquatic organisms as well as human health. Such effects include genetic alterations and physiological modifications, and in extreme cases, death of the exposed organism. However, the effects are less noticeable in low applications of ATZ but can, ultimately, reduce their life expectancy [6]. Fish can play a role in estimating the risk associated with ATZ as they are directly or indirectly exposed through the food chain of the environment or surface running water [10]. Some herbicides can induce oxidative stress expressing with excess production of ROS [11,12] that could be neutralized by antioxidant compounds and antioxidant enzyme system (AOS).

In humans, exposure to ATZ is common in farm workers and herbicide applicators, who are frequently exposed to it. Such herbicides can enter the body through inhalation as well as swallowing food, water, or soil that contain ATZ. On the other hand, it does not easily pass through the skin. Once ATZ enters the systemic circulation, it is converted into metabolites and can enter some of the organs, such as the liver, ovary, kidneys, red blood cells, or fat. It does not stay in the body and usually gets excreted primarily in the urine and a small amount through feces within 1 to 2 days [13]. A prospective cohort study found a positive association with multiple myeloma, non-Hodgkin lymphoma, and cancers of the bladder and lung [14]. In contrast, another study showed no relationship between ATZ use and most of the cancer site [15]. Furthermore, a case–control study found a possible association between non-Hodgkin lymphoma and ATZ use in men after statistically adjusting for other pesticides [16]. Additionally, farmers exposed to triazine herbicides had an increased risk of having non-Hodgkin’s lymphoma (NHL) [17].

### 1.2. Effects of Atrazine on Various Systems of the Body

**Figure 1:**
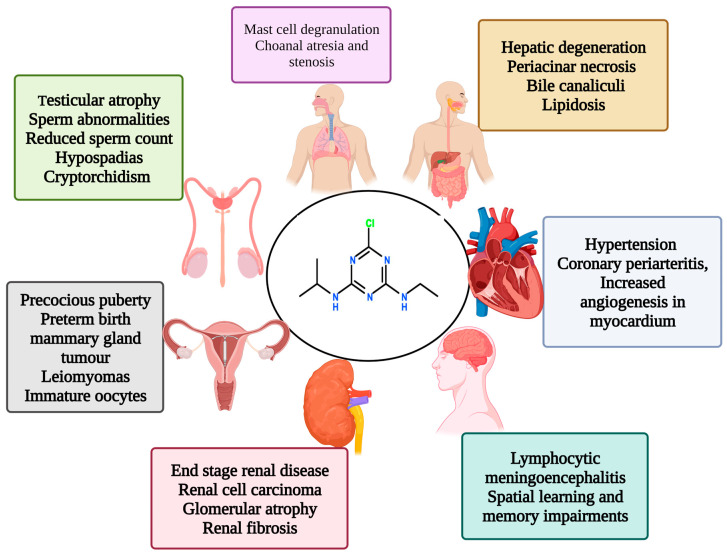
The adverse effects of atrazine exposure on various body systems.

ATZ effects are seen in most systems, and its effects on the reproductive, excretory, and nervous systems were extensively investigated. The effects of ATZ on various body systems are shown in Figure 1.

Musculoskeletal system

ATZ causes functional and morphological changes in the skeletal muscle. Chronic administration of ATZ in rats caused a decrease in basal metabolic rate, an increase in intra-abdominal fat deposition, further swollen mitochondria in skeletal muscle and liver, and a loss of normal cytoarchitecture [18].

Reproductive system

ATZ affects the male and female reproductive organs, thereby decreasing the reproduction capacity. ATZ showed estrogenic and anti-androgenic activity [19]. ATZ exposure caused the disruption of claudin-11 and connexin-43 proteins in the blood testis barrier, and also affected the spermatocytes, thereby reducing the number of spermatids in the rat seminiferous tubule culture model [20]. Male rats exposed to ATZ showed irreversible testicular and seminiferous tubule atrophy, as well as a reduction in Leydig cell number [21]. Chronic ATZ exposure in mice caused reduced sperm concentration, and changed gene expression related to androgen conversion in the testis [22]. Decreased sperm count, testosterone level, sperm motility, and epididymal weight were observed in rats treated with ATZ [23]. Exposure to low doses of ATZ significantly increased the dead spermatozoa and decreased the sperm motility [24]. ATZ-received rats showed a decrease in daily sperm production, sperm motility, and epididymal and testicular sperm counts [25].

Prenatal ATZ exposure caused an increase in abnormal sperm counts; in addition, testicular and epididymal weights were decreased in postnatal exposure [26]. Mice prenatally exposed to ATZ showed defects in penile morphology, hypospadias, cryptorchidism, decreased anogenital distance, and penis size [27]. ATZ induced testicular toxicity, in the form of reduced daily sperm production, count, and motility and an increased dead/live sperm ratio, is associated with testicular oxidative stress [28]. ATZ-treated male mice showed testicular damage, reduced spermatogenesis, and apoptotic spermatocytes [29]. In utero exposure to ATZ caused demasculinization of male reproductive structures and increased the incidence of hypospadias in male mice [30].

Female rats treated with ATZ showed an increase in the estrogen to androgen ratio [31]. ATZ exposure in adult ovariectomized Wistar rats decreased the GnRH neurons, thereby significantly reducing the FSH and LH hormone surges [32]. ATZ administration for 14 days markedly reduced the peak LH surge in rats [33]. In a multigenerational study, ATZ exposed rats of the F1 generation did not show any adverse effects [34]. However, F2 generation animals showed testicular diseases, precocious puberty in males, and mammary gland tumors in both males and females. Precocious puberty in female animals, motor hyperactivity, and a high frequency of testis disease were observed in F3 generation animals [34]. ATZ exposure altered the estrous cycle activity in female Wistar rats. Vaginal opening was delayed, and it was not observed in a few animals. Estrous cycle was irregular with a longer phase of diestrus [35]. Low-level ATZ exposure showed more defects in chromosomal synapsis, less quality of oocytes, and structural and numerical abnormalities of chromosomes [36]. ATZ exposure in female mice reduced the number of primordial follicles, increased the number of multi oocyte follicles, and further interfered with prophase I of meiotic division, which affects the follicle maturation process [37]. In utero exposure to ATZ showed endometrial hyperplasia and leiomyomas in female mice [38]. In a recent study, Multi-Generational atrazine exposure in mice altered early steroidogenesis gene expression in F1 and F2 generations and germ cell-specific gene expression in F1 [39]. Furthermore, preterm births increased in people living in the ATZ exposure counties [40]. Women exposed to ATZ affected the steroidogenesis and ovulation in cumulus granulosa cells, compromising female reproduction [41].

No association between preterm birth and ATZ exposure due to public drinking was observed in the Western region of Kentucky [40]. Another study in France reported an association between the high ATZ levels in drinking water and preterm birth [42]. Another study conducted in the Indiana region showed a positive link between ATZ exposure and infants who are small for-gestational-age [43]. Researchers also reported an association between ATZ exposure and intrauterine growth inhibition in the Iowa region [44].

Gastrointestinal system

ATZ toxicity is seen in the liver. Rats receiving oral ATZ showed oxidative stress, degeneration, and apoptosis in submandibular salivary glands [45]. ATZ-treated rats showed degenerated hepatocytes with markedly high levels of ALP, AST, and ALT total bilirubin concentrations and reduced GSH [45]. Rats exposed to even the smallest doses of ATZ showed portal lymphocytic inflammation, hepatic peri-acinar necrosis, and lipidosis in hepatocytes [46]. Subacute exposure to ATZ led to an increase in catalase, SOD, and GST in the liver; in addition, genotoxicity was also observed [47]. In a recent review, it was concluded that ATZ exposure causes oxidative stress and alters the expression of genes that are linked to hepatocyte function [48]. Repeated ATZ exposure enhanced the GST expression, and mRNA expression levels of various cytochromes; however, chronic administration caused habituation or adaptation [49]. ATZ elevated Na(+)-K(+)-ATPase activity and diminished Mg(2+)-ATPase and Ca(2+)-Mg(2+)-ATPase activities in mouse liver [50]. Following ATZ administration, an electron microscopic study of rat liver revealed dose-dependent histopathological changes such as smooth ER degeneration, lipid accumulation in hepatocytes, and structural alterations in bile canaliculi [51]. ATZ was found to affect the small intestine, it decreased the epithelial height of intestinal villi, ratio between villus height to mucosa thickness, and crypt depth, whereas high doses of ATZ significantly increased these features in the rat jejunum [52]. In an in vitro study, ATZ enhanced the cell proliferation in human colonic epithelial cells [53].

Respiratory system

Inhalation is one of the main routes of ATZ entry into the body, it affects the respiratory system. ATZ aerosol inhaled mice showed that oxidative and nitrosative stress increased cytokines and lipid peroxidation, and apoptosis that resulted in enhanced mucus production and mast cell degranulation [54]. Furthermore, in humans, ATZ exposure increased the risk of developing choanal atresia and stenosis [55].

Nervous system

ATZ has toxic effects on the central nervous system. ATZ-treated rats showed degeneration and apoptosis in the cerebrum and hippocampus [45]. Rats exposed to even lower doses of ATZ showed lymphocytic meningoencephalitis [46]. ATZ-treated rats showed degenerated, vacuolated neurons in the cerebellum [56]. Male mice treated with ATZ showed oxidative stress, inflammatory damage such as neuronal swelling, and mitochondrial vacuolar degeneration [57]. Maternal ATZ exposure caused spatial learning and memory impairments and hippocampal damage in offspring rats [58]. ATZ exposure in rats damages the hippocampus and affects spatial memory. It also downregulated the dopamine receptors [59].

ATZ exposure degenerated the nigrostriatal dopaminergic neurons, leading to the effects on motor functioning behavior [60]. ATZ exposure through drinking water during gestation and lactation damaged the nigrostriatal dopaminergic pathway, resulting in functional changes in motor and emotion in juvenile offspring and decreased cognition in adult offspring [61]. Pregnant rats exposed to ATZ decreased the dopamine concentration in their offspring [62]. Chronic ATZ exposure showed changes in the nigrostriatal dopaminergic pathway such as hyperactivity, decreased dopamine levels, increased anxiety, and extracellular glutamate levels in the striatum [63]. Sprague Dawley male rats exposed to ATZ showed an increase in the GABAergic neuron gene expression in the striatum and ventral midbrain, glutamatergic neuron expression was found in hippocampus [64]. ATZ caused hypoactivity soon after its administration, it significantly decreased locomotor activity [65]. Short term ATZ exposure in mice showed behavioral changes, motor and cognitive function impairments, and elevated anxiety [66]. In an in vitro study, ATZ inhibited the growth of human embryonic stem cells and neural stem cells [67].

Cardiovascular system

ATZ induces cardiotoxicity in the form of enhanced plasma total cholesterol, HDL-cholesterol, LDL-cholesterol, and triglycerides [28]. Maternal ATZ exposure in Sprague Dawley rats increased the blood pressure in both male and female offspring [68]. Rats exposed to even lower doses of ATZ showed coronary periarteritis [46]. In a study on isolated rat aorta and heart, ATZ caused vasodilatation of the aorta and significantly inhibited the normal twitch tension of isolated heart [69]. ATZ exposure in the mice decreased the creatine kinase activity, changes in the ionic content, and downregulation of sodium, potassium, and calcium ATPase activities [50]. Juvenile and peripubertal male Wistar rats exposed to ATZ showed an increase in angiogenesis in the form of enhanced numerical and volumetric density of capillaries in the left ventricle myocardium [70].

Endocrine system

ATZ disrupts the hypothalamic pituitary axis; hence, it is classified as an endocrine-disrupting chemical. ATZ-administered rats showed vacuolated follicular cells of the thyroid gland [56].

Excretory system

ATZ toxic effects are marked on the kidneys, as they are the clearance routes. Chronic ATZ exposure increases the risk of developing end stage renal disease [71]. ATZ exposure was found to be associated with an increased risk of renal cell carcinoma [72]. Short-term ATZ exposure resulted in elevated levels of antioxidant enzymes and kidney function biomarkers such as creatinine and urea [73]. ATZ administration in female Wistar rats caused increased serum urea nitrogen and creatinine levels; in addition, nitric oxide and malondialdehyde levels were also increased in kidney tissue homogenates [74]. ATZ-treated male mice showed histopathological changes and biochemical alterations by activating the nuclear xenobiotic receptors, disrupting cytochrome P450s homeostasis, and inducing nephrosis and renal injury [75]. ATZ exposure in male mice induces nephrosis and causes renal injury via activation of nuclear xenobiotic receptors [75]. ATZ caused renal injury in mice in the form of renal tubular epithelial cell edema and glomerular atrophy [76]. Wistar rats exposed to ATZ had increased renal peroxidative damage and serum uric acid levels [77]. Long-term ATZ exposure in rats caused the renal degenerative changes and fibrosis in rats [78].

ATZ-exposed human male pesticide applicators showed altered kidney function, chronic kidney disease, and a reduced glomerular filtration rate [79]. ATZ lowered eGFR and enhanced the risk of end stage renal disease as well as chronic kidney disease among male pesticide applicators [79]. ATZ applicators were found to have an increased incidence of renal cell carcinoma in later stages of life [72].

Integumentary system and blood

In a model of a flow-through in vitro diffusion system, absorption as well as metabolism of ATZ through human skin was examined. About 16.4% of the applied dose of ATZ was absorbed by the skin [80]. The same study concluded that skin microsomal enzymes are involved in the biotransformation of the ATZ [80].

In a research study, six volunteers received exposures through the skin and inhalation. It was found that the metabolism was rapid, with equal amounts of the deisopropyl metabolite and the fully N-dealkylated metabolite being produced [81]. In another study involving human skin, three-quarters of the ATZ applied was remained in the skin following 20 h, and 50% of the total metabolites such as deisopropylatrazine and diamino derivatives [82]. ATZ and its metabolites have toxic effects on the blood. The mice administered high doses of ATZ revealed atrophy and destruction of the thymus and spleen, it caused immunotoxicity through the cellular and humoral immunity pathways [83]. In addition to that, in utero ATZ exposure significantly decreased the clonogenic capacity of myeloid progenitor cells in male mice [84]. In fish (*Schizothorax plagiostomus*), ATZ exposure altered the biochemical and hematological parameters and promoted the DNA damage in erythrocytes [85].

## 2. Mechanism of Action of Atrazine

ATZ is a well-known endocrine disrupting compound. Its exposure affects the neuroendocrine system and associated endocrine axes, including the hypothalamus–pituitary–gonadal (HPG) axis and the hypothalamic–pituitary–adrenal (HPA) axis. The hypothalamus consists of abundant GnRH neurons that release GnRH [32,86]. ATZ affects the production of LH and FSH from the anterior pituitary by inhibiting the release GnRH. By changing the hypothalamic regulation of hormones, ATZ was reported to reduce the prolactin concentrations and the amplitude of the luteinizing hormone (LH) spike in experimental female Sprague Dawley and Long-Evans rats [87]. ATZ exposure increases *Kiss1* mRNA levels and decreases GnRH release, ultimately resulting in a reduction in anterior pituitary hormones: LH and FSH. Reduced amounts of these anterior pituitary hormones promote changes in estrogen, testosterone, and progesterone levels [88]. ATZ deleterious effects on the reproductive system are due to its action on the steroid synthesis [89]. ATZ exposure also reduced the expression of *Lhr* mRNA and the ovulatory genes *Areg*, *Ereg*, and *PgR* gene expression [90]. Furthermore, the ATZ-induced abnormal ovarian morphology and progesterone [91] are mediated by increasing the *Star* and *Cyp11a1* markers expression through ERK1/2, cAMP, AKT, and CREBPB-signaling pathways and inhibiting *phosphodiesterase 4* (*Pde4*) [91,92,93,94]. ATZ increases aromatase (*CYP19A1*) activity, increasing the aromatization of testosterone and its conversion to estrogen [93,95]. However, controversial results were reported on the effects of ATZ on *CYP19A1* [31,96].

**Figure 2:**
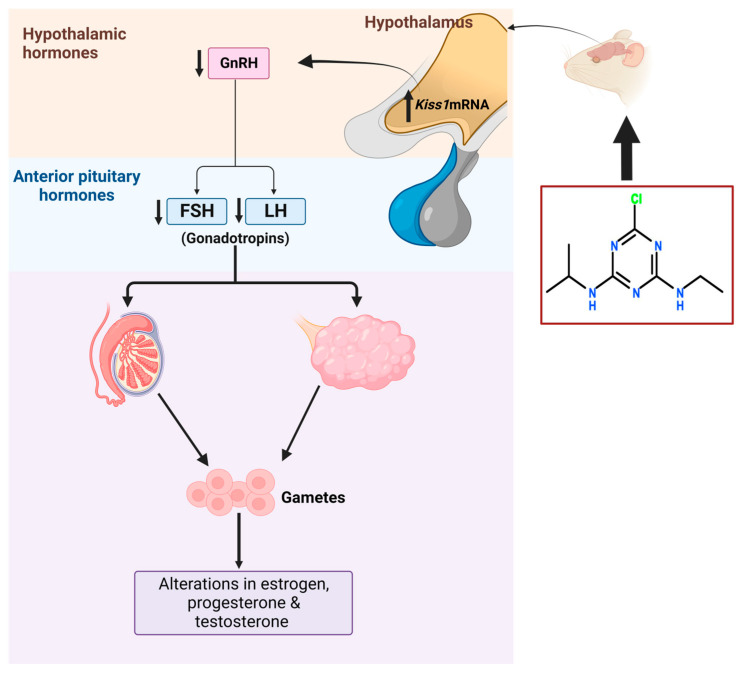
The mechanism of action of atrazine on male and female reproductive system. Atrazine exposure is associated with alterations in estrogen, testosterone and progesterone hormones through Kiss 1mRNA regulation.

The mechanism of action of ATZ on reproductive system is shown in Figure 2.

## 3. Approaches to Counteract Atrazine Toxicity

There are different approaches that target the elimination of ATZ toxicity in the environment, which include restricting the use of ATZ and the use of alternative methods that reduce reliance on ATZ. In an attempt to eliminate ATZ toxicity in the environment, the US Environmental Protection Agency implemented measures that result in reducing runoff, such as the use of terraces or vegetated filter strips, or producing more cover crops, or reducing the use of ATZ overall [97]. The number of measures taken by the US Environmental Protection Agency on growers varies depending on factors such as the concentration of ATZ in the watershed in the field, the likelihood of the watershed being exposed to ATZ, and the amount of ATZ used by the grower. The agency could require the use of some or all of these measures, or they could provide a picklist in which the grower could select the measures that would best suit him. This selection is based on factors such as the geographic region, field topography, and crop [97].

An alternative method to reduce ATZ concentration is by using adsorbents such as activated carbon, biochar, bentonite, and zeolite. However, activated carbon use may be limited due to its high cost, pollution, and difficult regeneration [98]. Biological treatment technology can also be used, which results in the degradation of ATZ. This includes microbial remediation, phytoremediation, and plant-microbial remediation. A study conducted by Sánchez et al. (2017) aimed to explore the ability of ryegrass, tall fescue, barley, and maize, to degrade ATZ via phytoremediation, and it showed that all of them had the ability to do so [99]. Alternatively, growers can reduce their reliance on ATZ in different ways: (1) by producing cover crops, which compete with weeds for nutrients, light, and water. However, it would increase the cost because the grower would essentially be placing and removing crops that do not result in any income. Additionally, it is not feasible for sweet corn because its seedling vigor is reduced in fields with cover crop residues, and it is not feasible for sugarcane because it is perennial [100]; (2) by using rotary hoes, which can cover a large area in a short period of time [101]; (3) postponing the use of a portion of fertilizers on corn crops until they are best able to absorb them can prevent weeds from getting the nutrients, they need to grow [102]; (4) through the use of a method called crop rotation, which involves growing different crops in the same area but in recurring sequences. This method prevents weeds from multiplying and reduces the likelihood of developing resistant weeds [103].

## 4. Natural Products and Compounds as Possible Agents to Counteract Atrazine Toxicity

Lycopene

The lycopene (Lyc) extraction could provide a food grade resource of carotenoid [104]. Its bioavailability depends on dietary content. Its consumption along with a fatty meal amplifies its bioavailability [105]. Lyc’s anti-cancer property is due to its ability to inhibit the cell cycle and induce apoptosis [106]. The cardio-protective property of Lyc has the ability to modulate several essential actions, including apoptosis and inflammation [107]. Neurobiological enhancing effects of Lyc were reported on various neurodegenerative diseases, including Parkinson’s and Alzheimer’s diseases [108].

The most important health challenges of the liver include hepatitis, cirrhosis, fibrosis, and liver carcinoma. One study discussed the hepato-protective properties of lycopene and also investigated the mechanisms behind this kind of effect [109]. Lyc is a structurally a carotenoid widely found in fruits and vegetables. It reduces oxidative stress. It showed a potential preventive role against ATZ-induced Nlrp3 inflammasome activation in spleen through ox-mtDNA depletion [110]. In mice, Lyc pretreatment inhibited ATZ-induced oxidative damage in the cerebrum via xenobiotic-sensing nuclear receptors and CYP450s modulation [57]. Lyc co-supplementation along with ATZ regulated the IL-6/STAT3/Foxo1 axis increased thymic CD45 levels and maintained thymic homeostasis [110].

Curcumin

Curcumin is a polyphenol that is prepared from the Curcuma aromatica Salisb root tuber and the rhizome of C. longa L. Curcumin, chemically known as 1,7-bis(4-hydroxy-3-methoxyphenyl)-1, 6-heptadiene-3, 5-dione. Its main biological activities include anti-inflammatory, anti-tumor, and anti-oxidant activities [111]. Its other activities are antidiabetic, anti-proliferative, antibacterial, antifungal, and anticancer, etc. [112] It shows anti-inflammatory effects through inhibition of interleukin-4 (IL-4), a pro-inflammatory cytokine secretion [113]. Curcumin can improve cardiovascular function by delaying cellular senescence, inhibiting the oxidative stress induced cell senescence and reducing ROS production [114]. Curcumin had positive, neuroprotective results on motor, sensory function as well as cognitive deficits [115]. Curcumin has therapeutic potential for the reproductive system by decreasing the risk of cancer and other malignant diseases [116]. In rats, curcumin supplementation showed significant cardiac protection against ATZ exposure associated cardiotoxicity via redox status modulation, improving the mitochondrial function and expression of caspase-3 [117]. Pretreatment with curcumin in rats against ATZ toxicity showed positive results by improving the anti-oxidant effect in hepato-renal injury [77]. In male albino rats, curcumin and ATZ co-supplementation prevented DNA lysis, oxidative damage, apoptosis, and mitochondrial dysfunction [118]. Curcumin administration in rats prevented the ATZ exposure-induced alterations in reproductive hormones and testicular injury [119].

*Panax ginseng* Essential Oil

*Panax ginseng*, popularly known as Asian ginseng, is a plant species that possesses antioxidant, immune stimulating, cardio protective, anti-aging, and anti-tumor properties [120]. Panax ginseng consists of functional bioactive compounds called ginsenosides. It has a wide range of properties such as anti-inflammatory, anti-allergic, and antidiabetic activities [120,121]. Supplementation of ginseng in regular diets is shown to boost the reproductive effectiveness of African catfish [121]. Panax ginseng essential oil (GEO) ameliorated the sub-lethal dose of ATZ induced toxicity in *Nile tilapia* fish. This study revealed that GEO supplementation in diet significantly improved the lipid metabolism and antioxidant status in the liver and enhanced immune function. These effects were mediated through its anti-apoptotic, antioxidative, anti-stress, and anti-inflammatory activities [122].

*Spirulina platensis*

Spirulina (*Spirulina platensis*) (SP) is a blue, green microalga, belonging to the cyanobacteria family, and it contains the majority of therapeutic and prophylactic components of nutrition [123]. Spirulina (*Spirulina platensis*) contains huge amounts of protein, fat, carbohydrate, chlorophyll, phycocyanin, vitamins, minerals, carotenoid, and other pigments that are favorable to health. It is an appropriate meal for humans and animals because its cell wall lacks cellulose [124]. The presence of the phycocyanin component in spirulina can promote anti-arthritic properties, and it has anti-atherogenic, chemo- and radio-protective, and tumor-inhibiting properties [125]. Studies concluded that *Spirulina* can suppress tumorigenesis and critical viral infections, apparently due to its capacity to stimulate and progress the immune system [126]. Several studies showed its neuroprotective properties of Spirulina against neuroinflammation, Parkinson’s disease, ischemic brain damage, and schizophrenia [127,128,129]. Oral administration of the phycocyanin component of *Spirulina platensis* can prevent the diabetic nephropathy by inhibiting NADPH dependent superoxide production [130]. In carps, *SP* showed beneficial effects against ATZ exposure induced oxidative stress and associated liver damage [131]. In *Cyprinus carpio* L, SP dietary intake prevented the ATZ exposure-induced immune responses [132]. An experiment on adult female zebrafish showed that SP supplementation ameliorates ATZ induced toxic effects across generations [133].

Fucoidans

Fucoidans are fucose-rich polymers that belong to the sulfated class, and they are found in various species of brown seaweed. They are known to be present in *Laminaria japonica* (kombu), *Cladosiphon* sp. (mozuku), *Undaria pinnatifida* (wakame), and *Fucus vesiculosus* (bladderwrack) [134,135]. Fucoidan has various biological activities, including anti-tumor and immune modulation properties [136], anti-coagulant effect [137] and anti-inflammatory effects [138]. The cardiovascular protective properties of fucoidan and its applications on the coagulation system, inflammation, and vascular cells were discussed in one study [139]. Its neuroprotective effects against brain injury, amyotrophic lateral sclerosis, Alzheimer’s disease, and Parkinson’s disease were reviewed [140]. Fucoidans supplementation in the mice mitigates the musculoskeletal changes and promotes muscle health and performance [141]. They suppress tumor cell proliferation factors and metastasis by increasing cell apoptosis and angiogenesis inhibition [142]. ATZ-treated fish revealed deterioration of the epithelium, intestinal mucosa, inflammatory cell infiltration, and enzyme values of the liver and kidney, but this condition was different and better in the fucoidan treated group, and this study showed dietary fucoidan is essential in fish diets to improve the impacts of ATZ induced toxicity [143].

Vitamin C

Vitamin C is well known as L-ascorbic acid; it is especially popular among the general population primarily due to its antioxidant properties. The sources of vitamin C are fruits such as star fruit, kiwi, guava, black currant, and strawberry. A sufficient amount of vitamin C is mainly present in the citrus family [144,145,146]. The average plasma levels of vitamin C in healthy adults are between 40 and 65 µM [147]. Various biological properties of vitamin C supplementation can prevent and treat diseases such as cardiovascular disease, cancer, inflammatory conditions, hematopoietic soft tissues, and behavioral impairments [148,149,150,151]. Vitamin C supplementation is beneficial against acute respiratory distress syndrome [152] and neurodegenerative diseases such as Alzheimer’s disease [153,154]. Sufficient supplementation of vitamin C in the diet of a poorly nourished population showed beneficial effects on cellular and DNA integrity [155]. An animal study revealed the protective role and anti-oxidant mechanism of vitamin C in kidney function and renal arterial reactivity against renal ischemia and reperfusion injury [156]. Vitamin C can reduce cardiovascular risk by decreasing the production of monocyte adhesion to the endothelium, and adhesions or atheromas, are considered early signs of the development of atherosclerosis [157].

ATZ herbicide exposure-induced toxicity was treated by dietary supplementation of vitamin C on *Rhamdia quelen* fish, and the results showed that vitamin C could reverse the abnormal liver biomarkers [158]. A study conducted on the degradation efficiency and the degradation mechanism of ATZ in the presence of vitamin C at different pH values by investigated by liquid chromatography, mass spectrometry, high performance liquid chromatography, and ion chromatography [159]. This study’s results help understand how vitamin C applications can solve organic pollutants [159].

Soybean isoflavones

Soybeans mainly contain isoflavones such as genistein, formononetin, daidzein, biochanin A, and coumestrol. They are easy to consume daily in the diet [160]. Chemically, isoflavones belong to the flavonoid family with a 3-phenylchromone skeleton [161]. According to the FDA, an intake of 50 mg of isoflavones per day is considered safe and sound [162]. Constituents of soybean isoflavones such as genistein and daidzein have anti-cancer and antioxidative effects, and also affect a variety of lifestyle diseases [163]. Genistein has proven neuroprotective effects by blocking the neurotoxicity in the nerve cells of the brain [164]. Genistein has anti-obesity properties that act directly on the adipocytes or preadipocytes and modulate obesity-related metabolic diseases [165]. Genistein also has antihypertensive properties through which it reduces the cardiac failure [166]. Genistein plays a vital role as an anti-inflammatory and anti-lipid peroxidase effect by regulating the gene expressions associated with liver inflammation and fibrosis [167]. Kidney dysfunction due to ischemia and reperfusion was improved by Genistein [168]. Soy isoflavones show anti carcinogenic effects by suppressing the expression of tyrosine kinase, apoptosis, and regulating the cell cycle [169].

Pre-treatment of SH-SY5Y neurons with soybean isoflavones prevented ATZ-induced metabolic failure and cytotoxicity [170]. In the same study, the soybean isoflavones prevented neurotoxicity and mitochondrial dysfunction by modulating the BEX2/BNIP3/NIX pathway [170]. Soybean isoflavones can prevent ATZ exposure induced DAergic neurons degeneration by mTOR-dependent signaling pathway mediated autophagy activation [171].

Quercetin

Quercetin, derived from quercetum (oak forest), is widely available in plants, normal vegetables, and leaves. It is also found in some medicinal plants such as elderberry, *Ginkgo biloba*, and *Hypericum perforatum* [172]. Quercetin is a highly antioxidant compound that can directly scavenge free radicals and inhibit lipid peroxidation activity [173]. The antimicrobial properties of Quercetin can fight various bacteria [174]. Quercetin can facilitate the mitochondrial synthesis, minimize protein or amino acid utilization, and improve energy [175]. Quercetin is an anti-tumor compound, this property is performed through by preventing the cell cycle process, promoting cell apoptosis, and reducing blood vessel generation and transfer [176]. Quercetin exerts anti-inflammatory activities on both endothelial cells and monocyte macrophages [177]. The cardioprotective activity of Quercetin showed positive beneficial effects on atherosclerosis, hypertension, and cardiotoxicity [178]. L-Carnitine plays a significant role in proteolysis, protein synthesis, and the maintenance of skeletal muscle protein balance [179]. L-Carnitine and Quercetin have a positive effect on promoting fatty acid oxidation [180]. Exercise-induced fatigue was ameliorated by Quercetin and L-Carnitine and also other compounds through targeting multi-signaling pathways in mice [181].

An experimental study in the adult male albino rat’s showed that significant protective action against the ATZ-induced reproductive toxicity was reversed in a dose-dependent manner such as Quercetin in low dose and L-Carnitine in both low and high doses [182]. Quercetin’s action against ATZ-induced testicular toxicity in an experimental animal study showed positive results, such as improved testicular function and partial improvement of sperm motility [183]. In one study, treatment with quercetin against sub-acutely induced ATZ toxicity in rats showed improved reproductive function and sperm quality through an antioxidant defense mechanism [184].

Fluted pumpkin seeds

Fluted pumpkin (*Telfairia occidentalis*) is a vegetable found in West Africa and cultivated for its edible seeds and leaves [185]. In addition to its nutritional value, it is widely used as a medicinal herb to treat many diseases such as diabetes, anemia, hypertension, and malaria [186]. Fluted pumpkin seeds (FPS) are rich in proteins, carbohydrates, minerals, and vitamins. Moreover, FPS are rich in bioactive constituents that possess different biochemical and physiological effects [187]. A study demonstrated that FPS have antioxidant, anti-cancer, and anti-inflammatory activities [188]. FPS were also used traditionally to improve sexual performance and enhance fertility in men [189]. FPS showed a protective potential against oxidative damage induced by chemotherapy in germ cells [190]. In addition to the anti-oxidant property, FPS also possesses an anti-inflammatory effect through the inhibition of interleukin-6 and serum nitrite [191]. Due to its healing properties, FPS was traditionally used to treat various diseases in Asia and Africa [186].

FPS’s beneficial effects against ATZ exposure-induced toxicity is not well explored. Only one study demonstrated the protective effect of FBS against the toxicity of ATZ [192]. In this study, daily treatment of Wister rats with FPS extract (25 mg/kg) significantly checked the testicular damage induced by ATZ [192]. They found that FPS protected the testis by decreasing malondialdehyde and increasing glutathione concentrations in the testicular tissue. However, they found that the higher dose of FPS (50 mg/kg) was harmful to the testes [192].

Vitamin E

Vitamin E is a fat soluble antioxidant vitamin found in varied foods such as fruits, vegetables, meat, and eggs [193]. Vegetable oils are the main source of vitamin E. It consists of two main groups, namely, tocotrienols and tocopherols, which are known as effective antioxidants [193]. Each of these groups are also divided into alpha, beta, gamma, and delta isomers. A previous report showed that vitamin E has a potent antioxidant and anti-inflammatory properties, which are highly beneficial in different aspects of health [194]. It was also reported that vitamin E has the ability to neutralize peroxyl radicals and lipid peroxidation. The antioxidant activity of vitamin E can protect the polyunsaturated fatty acids in the cell membrane by removing reactive oxygen species and reactive nitrogen species [195,196,197,198].

Studies showed that vitamin E has a protective effect against ATZ-induced genotoxicity in liver cells. Administration of vitamin E (300 mg/Kg) significantly attenuated the DNA damage induced by ATZ [199,200]. A study revealed that the co-administration of vitamin E and testosterone significantly ameliorated the toxic effects of ATZ on sperm quality and testis by increasing endocrine function and antioxidant capacity [201]. The antioxidant property of vitamin E reduced apoptosis and increased steroidogenesis of Leydig cells. Moreover, the oxidative stress induced by ATZ in shrimp palaemonetes argentines was remarkably decreased by the antioxidant effect of vitamin E [202]. In another in vitro study, vitamin E was able to delay the ATZ-induced degenerative changes of goat testicular tissue [203].

Garcinia kola seeds

*Garcinia kola* (GK) is a plant found in Asia and tropical Africa and is also known commonly as bitter cola because of the bitter taste of its seeds [204]. GK stems, roots, and seeds were traditionally used to treat diabetes mellitus, liver disorders, and sickle cell disease [205,206]. GK has anti-inflammatory, antioxidant, and antimicrobial properties [207]. The pharmacological properties of GK seeds are attributed to their complex mixture of biflavonoids, polyphenolic compounds, and prenylated benzophenones. Kolaviron is one of the biflavonoids that are extracted from GK seeds and is known as the most active phytochemical in these seeds [208]. Normal consumption levels of GK seeds are considered safe for human [207]. Recent reports demonstrated that GK attenuated oxidative stress through enhancing the antioxidant enzymes and suppressing the inflammatory markers [209]. Thus, GK seeds are potent remedy to restore kidney, liver, and testicular markers [210].

An in vitro study showed that kolaviron biflavanoids (KB) of GK seeds attenuated ATZ-induced cytotoxicity of cultured interstitial Leydig primary cells (ILCs) [210]. Treatment with KB protected ILCs by decreasing the levels of malondialdehyde (MDA) and reactive oxygen species (ROS). KB has significantly restored the expression of the steroidogenesis gene to normal in ILCs exposed to ATZ [211]. KB are known to possess anti-apoptotic activities. Treatment of PC12 cells by KB significantly reduced the apoptosis induced by ATZ. Inhibition of apoptosis in PC12 cells was achieved through the downregulation of ROS, malondialdehyde, caspase-3 activity, and the increase in glutathione and catalase activity. The expression of apoptosis markers such as caspase-3, caspase-9, and p53 was also restored by KB treatment [212].

Melatonin

Melatonin (N-acetyl-5-methoxytryptamine) is a natural indolic compound derived from tryptophan widely found in different organisms, including bacteria and eukaryotes [213]. Melatonin is also found in aromatic plants, leaves, and seeds. In mammals, pineal gland produces melatonin as its main secretory product. Melatonin, a known hormone or neurotransmitter, plays a role in various functions, including antioxidant and anti-inflammation activities and circadian rhythm regulation [214]. Melatonin is also synthesized in other organs, including, the brain, lungs, spleen, liver, kidney, and pancreas [215]. Many beneficial results of melatonin were obtained from experimental and clinical trials, which suggest using melatonin as a therapeutic drug to wield varieties of diseases such as neurological diseases, insomnia, and sleep disturbance [216,217,218].

**Figure 3:**
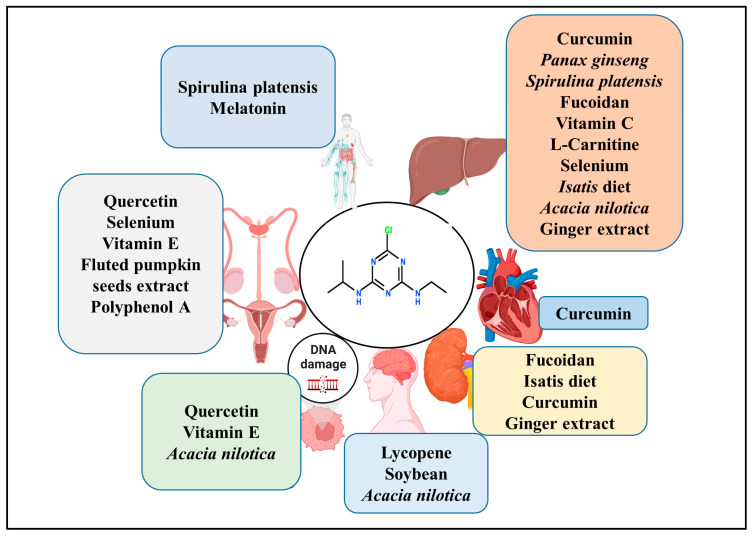
Schematic diagram showing the various natural compounds or natural products evaluated for their beneficial role against atrazine exposure-induced toxicity.

A potential role of melatonin on ATZ exposure-induced oxidative damage in rat erythrocytes was studied [219]. The study results revealed that melatonin supplementation significantly restored ATZ-induced morphological and biochemical changes in erythrocytes by scavenging the free radicals, activating superoxide dismutase and restoring the ATPases activity [219]. Another study showed that melatonin can inhibit ATZ induced-apoptosis by attenuating endoplasmic reticulum stress, Fas-mediated caspase 8 and 3 activation, and p53 independent mitochondrial apoptosis [220]. Figure 3 shows the various natural compounds that were studied against various toxic effects.

L-carnitine

Levocarnitine (L-Carnitine; LC) is an essential natural, water soluble compound for humans that can be found in most body tissues. It can be obtained from foods such as milk and meat and is also synthesized in the body including the brain, liver, and kidney [221]. LC plays an important role in lipid metabolism by working as an active amino acid derivative, micronutrient and facilitating long-chain fatty acid transportation into the mitochondria [222]. The protective effects of LC are due to its antioxidant properties which work as scavengers of reactive oxygen species [223]. In addition, LC reduces lipid peroxidation by facilitating the transport of long-chain fatty acids into the mitochondria to generate ATP for the cell [224]. It was demonstrated that LC could also repair oxidative damage and regenerate endogenous antioxidant activity [225,226].

Co-supplementation of LC at low and high doses (200 and 400 mg/kg body weight, respectively) significantly ameliorated the ATZ-induced reproductive toxicity [182]. In this study, LC was able to abolish the toxic effect of ATZ through the improvement of CYP17A1 mRNA, the indicators of serum oxidative stress, and serum testosterone [182]. In another study, it was shown that LC attenuated ATZ-induced hepatotoxicity in albino rats. LC significantly alleviated the hepatotoxicity by reducing inflammation, oxidative stress, and apoptosis in the liver [227]. The co-administration of LC restored the normal histological structure and improved antioxidant enzymes [227]. These studies demonstrated that LC has an ameliorative effect on ATZ-induced reproductive toxicity and hepatotoxicity through its anti-inflammatory, antioxidant, and anti-apoptotic properties [182,227].

Selenium

Selenium is an essential micronutrient that can be found naturally in water, soil, and air. It is important for various metabolic processes in the body. Selenium and its compounds can be introduced into humans through the raw materials of animals and plants [228]. It works as a cofactor enzyme that provides an anti-oxidative protection and regulates the inflammation in the body [229]. Selenium is an important component of glutathione peroxidase, an enzyme that protects membrane lipids from oxidative degradation [230]. A deficiency in selenium is associated with many adverse health effects, including viral infection, Kashin–Beck disease, autoimmune disease, and cardiovascular disease [231,232,233,234]. A high intake of selenium can change the composition of the gut microbiota [235]. Hepatic Selenoprotin P works as a survival factor by transporting proteins in the plasma to different parts of the body, including the brain [236].

Few experiments were conducted on the protective effect of selenium intake on ameliorating the damage induced by ATZ. A study by Adesiyan et al. (2011) evaluated the protective effect of selenium against ATZ-induced hepatotoxicity and reproductive toxicity in rats [25]. Selenium supplementation had no ameliorative effect against the biochemical changes that were induced by ATZ treatment in the testes [25]. However, selenium showed a protective effect against ATZ-induced biochemical alteration in the liver [25]. Another study evaluated the capacity of diphenyl diselenide (PhSe)_2_, an organo-selenium compound, to protect fish from damage induced by ATZ [237]. This study showed that diet supplementation of (PhSe)_2_ can protect cyprinus carpio (carp) against ATZ-induced damage through enhancing the activities of antioxidant enzymes [237].

*Isatis indigotica* (Cruciferae)

*Isatis* phytogenic extract has numerous functional components such as carotenoids, glycoproteins, polysaccharides, phenols, and essential oils [238]. *Isatis* is widely cultured in some European, Asian, and Middle Eastern regions. Isatis root powder is extracted primarily from *Isatis indigotica* (Cruciferae) [239]. In vivo and in vitro experiments concluded the anti-oxidant, anti-inflammation, antibacterial, antiviral, anti-cancer, and immunomodulatory effects of *Isatis* phytogenic extract [240,241]. *Isatis* leaves contain the highest content of indigotin, and indirubin which perform antibacterial activity [242]. ATZ exposure caused alterations in oxidative stress, immunity, and genotoxicity, subsequently leading to growth rate inhibition and feeding difficulties *Nile tilapia* [243]. In *Nile tilapia*, ATZ exposure-induced hepato-renal dysfunction, growth inhibition, and oxidative stress are significantly reversed by 1% dietary supplementation of *Isatis* [244].

Polyphenols

Polyphenols are widely found in edible plants. The main four major classes of polyphenols are the flavonoids, phenolic acids, stilbenes, and lignans [245]. Quercetin and kaempferol are the flavonols commonly present in fruits and vegetables [246]. Lignans are allicin derivatives, and this content is widely found in linseeds, sesame seeds, lentils, cabbage, pears, and garlic [247]. Stilbenes are not commonly found in food, but resveratrol and its derivatives are present in red grapes and red wine [248]. Lentils containing polyphenols showed a potential role in reducing blood pressure by inhibiting the angiotensin I-converting enzyme (ACE) activity in spontaneously hypertensive rats [249]. Studies concluded that polyphenols have chemo-preventive effects by modulating cancer cell signaling pathways and promoting apoptosis [250,251].

ATZ and its metabolites have a toxic effect on sperm quality due to the production of oxidative stress in the male reproductive system, and they also lead to a reduction in the fertilization competence of spermatozoa by impairing their morphology and altering the mitochondrial membrane potential [252]. In male goats (Capra hircus), dietary treatment with polyphenol prevented the ATZ exposure-induced changes in spermatozoa [252].

*Acacia nilotica*

*Acacia nilotica* is also called gum Arabic kikar and black babul. In Sudanese folk medicine, it is popularly called as ‘Garad’ or Sunt. It is a medicinal plant belonging to the Fabaceae family and is usually found in tropical and sub-tropical regions [253]. All parts of this plant were shown to be effective against various ailments such as neurological problems, tuberculosis, small pox, and GIT problems [254]. In the folk medicine of Sudan, it is used to strengthen teeth and reduce toothache [254]. The extract of *Acacia nilotica* has various phytochemical components such as flavonoids, tannins, and phenols [253]. *Acacia nilotica*’s potential role in treating diabetes mellitus, cancers, and inflammatory diseases was demonstrated. These effects were attributed to its antioxidant properties [255]. In a recent study, its seedpod was shown to possess anti-ulcerogenic activity [256]. Administration of 400 mg/kg/day of *Acacia nilotica* was partially protected against 200 mg/kg bw/day ATZ-induced toxicity by reversing the significantly elevated serum levels of AST, ALT, ALP, and decreasing GSH level in adult male albino rats [56].

*Zingiber officinale Roscoe*

*Zingiber officinale* Roscoe, popularly called ginger, belongs to the *Zingiberaceae* family. Traditionally, its underground rhizomes are used in food preparation as a spice [257]. It is a well-known herbal medicine and is frequently used as a home-remedy for treating various diseases including nausea, headache, common cold, and emesis. Its principal bioactive compounds are paradols, gingerols, shogaols, and terpene compounds [258]. Its potential biological activities include antioxidant, anti-inflammatory, antimicrobial, and anticancer activities [259,260]. Previous studies showed its potential ameliorative effects against neurodegenerative and cardiovascular diseases, metabolic syndrome, and respiratory disorders [260]. Its beneficial effects such as potent anti-platelet, antioxidant, anti-tumor, anti-rhinoviralis, anti-hepatotoxicity, and anti-arthritic activities are also demonstrated [261,262,263]. 

In mice, ATZ exposure-induced oxidative stress in both liver and kidney [264]. ATZ significantly in reduced the antioxidant enzymes activities and increased the lipid peroxidation [264]. The ginger co-supplementation on each alterative day for 14 days prevented the ATZ exposure-induced oxidative stress in liver and kidney [264]. These findings indicate that ginger can be used as a therapeutic agent to prevent ATZ-induced oxidative damage.

The effects of natural compounds against atrazine exposure-induced toxicity that were studied in various studies are depicted in Table 1.

## 5. Conclusions

The herbicide ATZ has potential toxic effects which are harmful to the human body. Admittedly, the use of herbicide cannot be curtailed but individuals handling such herbicide need to be educated about its toxic effects. In oxidative stress, there is also a disturbance in the antioxidant defense mechanism of the body and various enzymes which act as scavengers. ATZ exposure may result in a state of oxidative stress with the accumulation of free radicals. The increase in free radicals results in damage to the cells, RNA, DNA, lipids, carbohydrates, and proteins in the body. Oxidative stress plays an important role in various diseases related to cardiovascular, endocrine, central nervous, gastrointestinal, urinary, and reproductive systems. In future, potential biomakers could help us with warning signs of ATZ exposure. In the absence of any particular drug being available to treat atrazine toxicity, the only other option is trying natural compounds obtained from lycopene, curcumin, *Panax ginseng*, *Spirulina platensis*, Fucoidans, vitamin C, soyabeans, quercetin, L-carnitine, *Telfairia occidentalis*, vitamin E, *Garcinia kola*, melatonin, selenium, *Isatis indigotica*, polyphenols, *Acacia nilotica*, and *Zingiber officinale* for the treatment of toxicity. The phytochemicals such as flavanoids, tannins, alkaloids, and polyphenols compounds present in the natural products may act as antidotes to counteract the ATZ exposure induced toxicity. In the present review, we explored the need of future drug design based on evidence-based literature related to natural products and natural compounds which could be used to treat ATZ toxicity. Additionally, more studies required to explore the molecular mechanisms behind the ameliorative potential of natural products and also to identify the active compounds in these products/extracts. Further clinical trials are needed to develop a potential therapeutic drug to treat ATZ exposure-induced toxicity.

## Figures and Tables

**Table 1:** Effect of natural compounds or natural products against atrazine exposure-induced toxicity.

Author; Year	Animal Model	Atrazine Dose and Duration of Treatment	Natural Product or Natural Compound Dose and Duration of Treatment	Atrazine Induced Toxicity	Mechanism of Actions
Dai et al., 2022 [57]	Mice	50 and 200 mg/kg b.wt./day for 21 days	Lycopene—5 mg/kg b.wt./day for 21 days	Neurotoxicity	Anti-oxidant effect by modulating xenobiotic-sensing nuclear receptors and cytochrome P450
Keshk et al., 2014 [117]	Rats	400 mg/kg b.wt./day for 3 weeks	Curcumin—400 mg/kg b.wt./day for 3 weeks	Cardiac toxicity	Modulating redox status, mitochondrial function, caspase-3 expression
Abo El-Noor et al., 2014 [118]	Rats	100 mg/kg b.wt./day for 21 days	Curcumin—400 mg/kg/day for 21 days	Nephrotoxicity	By ameliorating the oxidative stress, apoptosis, DNA damage, mitochondrial dysfunction.
Ahmed et al., 2022 [122]	*Nile tilapia* (*Oreochromis niloticus*)	1.39 mg/L for 60 days	*Panax ginseng* essential oil—60 days	Growth inhibition and hepatotoxicity	Anti-oxidant and anti-apoptotic effects
Toughan et al., 2017 [131]	*Cyprinus carpio* L.	428 μg/L for 40 days	Spirulina (*Spirulina platensis*)—1% for 40 days	Hepatotoxicity	Anti-oxidant and anti-inflammatory effects
Khalil et al., 2017 [132]	*Cyprinus carpio* L.	428 μg/L for 40 days	Spirulina (*Spirulina platensis*)—1% for 40 days	Immunotoxicity	Immune related genes expression modulation and anti-inflammatory effect
Hedayatirad et al., 2020 [133]	Adult female Zebra fish	5 μg/L and 50 μg/L for 28 days	Spirulina (*Spirulina platensis*)—10 g/kg b.wt./day for 28 days	Immunotoxicity and endocrine disruptor toxicity	Transgenerational antimicrobial effects and immunotoxic suppression
Abdel-Warith et al., 2021 [143]	*Nile tilapia* fish	1/5 96 h LC_50_ (1.39 mg/L) for 30 days	Fucoidan—0.8% for 30 days	Growth retardation, hepatic and renal toxicity	Anti-oxidant and anti-inflammatory effects
Gomes et al., 2022 [158]	*Rhamdia quelen* fish	10 µgL^−1^ for 96 h	Vitamin C—1 g/kg b.wt for 30 days	Hepatotoxicity	Antioxidant and anti-peroxidase effect
Li et al., 2019 [171]	Rats	50 mg/kg for 45 days	Soybean—isoflavones 10, 50, or 100 mg/kg for 45 days	Neurotoxicity	Autophagy modulation through mTOR-dependent signalling pathway
Abdel Aziz et al., 2018 [182]	Rats	120 mg/kg b.wt. 21 days	Quercetin—10 and 50 mg/kg b.wt/day L-carnitine—200 and 400 mg/kg b.wt for 21 days	Reproductive toxicity and genotoxicity	Anti-oxidant effects
Abarikwu et al., 2016 [183]	Rats	120 mg/kg b.wt./day for 16 days	Quercetin—10 mg/kg b.wt./day for 16 days	Testicular toxicity	Anti-oxidant effects
Farombi et al., 2013 [184]	Rats	120 mg/kg/b.wt./day for 16 day	Quercetin—20 mg/kg/b.wt/day for 16 days	Testicular toxicity	Anti-oxidant effects
Abarikwu et al., 2022 [192]	Rats	50 mg/kg b.wt./day 60 days	Fluted pumpkin seeds extract—25 and 50 mg/kg b.wt/day for 60 days	Testicular toxicity	Antioxidant activity
Singh et al., 2008 [199]	Rats	300 mg/kg b.wt./day for 7,14 and 21 days	Vitamin E—100 mg/kg b.wt/day 7, 14, and 21 days	Genotoxicity	Antioxidant activity
Agdam et al., 2017 [201]	Rats	200 mg/kg b.wt./day for 22 and 48 days	Vitamin E—150 mg/kg/b.wt/day	Testicular toxicity	Promoting antioxidant capacity and endocrine function
Griboff et al., 2014 [202]	Shrimp Palaemonetes argentinus	0.4 mg/L for 21 days	Vitamin E—(16 mg%) for 21 days	Oxidative stress	Antioxidant effects
Bhatti et al., 2011 [219]	Rats	300 mg/kg of bw/day for 21 days	Melatonin—10 mg/kg bw/day for 21 days	Erythrocytes toxicity	Antioxidant effects
Sharma et al., 2014 [220]	Mice	100 mg/kg b.wt./day for 14 days	Melatonin—20 mg/kg b.wt/day for 14 days	Immunotoxicity	Suppression of endoplasmic reticulum stress, Fas-mediated and p53 independent mitochondria-mediated apoptosis and autophagy modulation
Rashad et al., 2023 [227]	Rats	400 mg/kg b.wt./day for 14 days	L-Carnitine—100 mg/kg b.wt/day for 14 days	Hepatotoxicity	Antioxidant, anti-inflammatory, and anti-apoptosis activities
Adesiya et al., 2011 [25]	Rat	120 mg/kg b.wt./day for 16 days	Selenium—0.25 mg/kg b.wt/day for 16 days	Hepatotoxicity	Antioxidant effects
Marins et al., 2018 [237]	Fish	2 or 10 µg/L for 96 h	Selenium compound diphenyl diselenide (PhSe)_2_ containing diet—3 mg/kg b.wt/day	Hepatotoxicity and reproductive toxicity	Antioxidant effects
Ali et al., 2021 [244]	*Nile tilapia* fish	1.39 mg/L for 30 days	Isatis diet—1% for 30 days	Hepatotoxicity and renal toxicity	Antioxidant effects
Komsky-Elbaz et al., 2019 [252]	Male goat (*Capra hircus*)	15 mg/kg b.wt./day for 6 months	Polyphenol A— standard ration for 90 days	Testicular toxicity	Antioxidant effects
Ahmed et al., 2022 [56]	Rats	200 mg/kg b.wt/day for 30 days	Acacia nilotica—400 mg/kg/day for 30 days	Hepatotoxicity, neurotoxicity and genotoxicity	Antioxidant effects
El-Shenawy et al., 2011 [264]	Mice	78.25 mg/kg b.wt./day for 14 days	Ginger extract—120 mg/kg b.wt for 14 days	Hepatotoxicity and renal toxicity	Antioxidant effect

## Data Availability

Not applicable.
